# Common pitfalls of stem cell differentiation: a guide to improving protocols for neurodegenerative disease models and research

**DOI:** 10.1007/s00018-016-2265-3

**Published:** 2016-05-06

**Authors:** Martin Engel, Dzung Do-Ha, Sonia Sanz Muñoz, Lezanne Ooi

**Affiliations:** Illawarra Health and Medical Research Institute, School of Biological Sciences, Faculty of Science, Medicine and Health, University of Wollongong, Wollongong, NSW Australia

**Keywords:** iPS cells, Differentiation, Neurodegeneration, Alzheimer’s disease, Dopaminergic neurons, Cholinergic neurons, Astrocytes

## Abstract

Induced pluripotent stem cells and embryonic stem cells have revolutionized cellular neuroscience, providing the opportunity to model neurological diseases and test potential therapeutics in a pre-clinical setting. The power of these models has been widely discussed, but the potential pitfalls of stem cell differentiation in this research are less well described. We have analyzed the literature that describes differentiation of human pluripotent stem cells into three neural cell types that are commonly used to study diseases, including forebrain cholinergic neurons for Alzheimer’s disease, midbrain dopaminergic neurons for Parkinson’s disease and cortical astrocytes for neurodegenerative and psychiatric disorders. Published protocols for differentiation vary widely in the reported efficiency of target cell generation. Additionally, characterization of the cells by expression profile and functionality differs between studies and is often insufficient, leading to highly variable protocol outcomes. We have synthesized this information into a simple methodology that can be followed when performing or assessing differentiation techniques. Finally we propose three considerations for future research, including the use of physiological O_2_ conditions, three-dimensional co-culture systems and microfluidics to control feeding cycles and growth factor gradients. Following these guidelines will help researchers to ensure that robust and meaningful data is generated, enabling the full potential of stem cell differentiation for disease modeling and regenerative medicine.

## Introduction

Studying mechanisms and potential therapeutic targets of neurodegenerative diseases has historically been limited by the timely access to the affected tissue. Furthermore, the use of animal models for disorders without a clear genetic cause has shown to be of limited translational value to the clinical setting. The possibility of creating neuronal cultures from human stem cells, particularly from induced pluripotent stem cells (iPSC) of diagnosed individuals, has received wide attention for the potential of creating translatable disease-in-a-dish models [1]. Following the discovery of iPSCs, several high profile publications have fuelled the enthusiasm for their use in research into Parkinson’s disease [2], Alzheimer’s disease [3, 4], motor neurone disease [5] and mental disorders [6].

While optimized methods for reprogramming iPSCs have broadly been adopted (recently reviewed by Revilla et al. [7]), the protocols for differentiating iPSCs into neuronal cultures vary significantly for the same desired cell types. Strategies for converting stem cells into terminally differentiated cells predominantly follow observations from developmental mouse and rat studies, intending to model the in vivo progression of chemical signaling. However, variations in composition, concentration and timing of the signaling molecules can lead to marked differences in the resulting cultures and maturation stage of the desired cells. Furthermore, recent studies report variations in the differentiation efficiency between different stem cell lines, which is particularly relevant if the differences are diagnosis-specific [8, 9]. Finally, novel culturing methods, including three-dimensional cultures and hypoxic conditions, have been reported to influence the differentiation efficiency [10, 11]. To ensure the generation of meaningful results, stem cell derived cultures will therefore need to undergo thorough characterization to identify the composition and functionality of the differentiated cells.

The development and maturation of neuronal cells depends on different types of chemical signaling in vivo. Initially, growth factors and chemoattractants released into the extracellular space by progenitor cells trigger location specific differentiation [12]. Upon neurite formation, local neurotransmitter release guides dendrite development and cell maturation to form the dynamic networks of the central nervous system [13, 14]. While growth factor signals can be recreated in vitro through time-dependent media supplementation, location and cell type-specific synaptic inputs cannot easily be modeled. It is therefore highly relevant to consider the developmental and maturation environment of the desired cell type when deciding to study disease processes in iPSC derived cultures.

With the present review, we provide an overview of existing differentiation strategies for neurodegenerative disease-relevant cell types of forebrain cholinergic neurons, midbrain dopaminergic neurons and cortical astrocytes. Advances in motor neuron differentiation have been extensively reviewed, including recently [15]. We address the variation in protocol efficiencies by providing a checklist that can be used to evaluate the quality and reproducibility of in vitro differentiation. Finally, we discuss recent culturing method developments aiming to improve the quality of stem cell derived neural cultures.

Human stem cell derived in vitro disease models have the potential to overcome the limitations of existing cell line work and can become a vital research stream next to animal modeling strategies. However, to progress in disease understanding and treatment development, the quality and biological relevance of stem cell-derived cultures need to be ensured.

## From in vivo development to in vitro differentiation

### Forebrain cholinergic neurons

Cholinergic neurons in the mammalian brain, including basal forebrain cholinergic neurons (BFCNs) and interneurons of the striatum, are defined by the production of acetylcholine (ACh) and its use as a neurotransmitter. BFCNs play an important role in cognitive functions, such as learning, memory and attention, and are implicated in the rapid eye movement sleep phase. The loss of BFCNs in neurodegenerative disorders, including Alzheimer’s disease, thus leads to severe cognitive impairments and memory deficits [16, 17].

#### Mammalian development of cholinergic neurons

During mammalian development, cholinergic neurons are derived from neural precursors of the ectoderm layer. After the formation of the neural tube, a selection of cells start to respond to high concentrations of sonic hedgehog (SHH), a morphogen that induces ventralization (differentiation towards the anterior) of the neural tube, and low concentrations of Wnt, a morphogen that induces caudalization (differentiation towards the posterior) [18]. The morphogens retinoic acid (RA), bone morphogenetic proteins (BMPs) and fibroblast growth factors (FGFs) are also known to play a key role in telencephalon development, the most anterior region of the developing brain [19]. In rodents and primates, BFCNs are formed in the medial ganglionic eminence (MGE), a ventral region of the telencephalon, with projections to the hippocampus and frontal cerebral cortex [20], areas implicated in the cognitive and psychological deficits of neurodegenerative diseases [21, 22].

The anterior/posterior patterning of the brain begins at embryonic day 8.5 (E8.5) in mice, when the telencephalic neuroepithelia expresses the transcription factors forkhead box G1 (FOXG1) and paired box 6 (PAX6) [23–25]. SHH together with FOXG1 induce the expression of FGFs, which activate downstream transcription factors characteristic of the ventral patterning. The expression of NK2 homeobox 1 (NKX2.1) at E9.5 defines the MGE region and by day E12.5 the expression of LIM homeobox 8 (LHX8) and insulin gene enhancer protein 1 (ISL1) determine a cholinergic fate. Choline acetyl transferase (ChAT) expression in neural precursor cells arises at postnatal day 7 (P7), with ChAT-positive cells increasing in number after P8 and remaining stable during adulthood [26].

While the full range of molecular drivers for BFCN development remains to be mapped, several essential factors for the differentiation, growth and survival of BFCNs have been identified. The cholinergic phenotype of embryonic BFCNs is induced and maintained by BMP9 [27], whilst FGF2 and brain-derived neurotrophic factor (BDNF) stimulate cholinergic differentiation [28, 29]. The maturation, neurotransmitter phenotype, arborisation and survival processes are finally controlled through nerve growth factor (NGF) signaling [30]. These growth factors drive the gene expression profile of BFCNs through specific transcription factors, such as LHX8 [31] and GBX1, a gastrulation box homeodomain protein, which can be used in the differentiation of BFCNs in vitro [32].

#### Characteristics of mature cholinergic neurons

Basal forebrain cholinergic neurons are characterized by their neuroanatomical location and the expression of ACh related genes. The key markers used to identify mature cholinergic neurons are involved in ACh synthesis (choline acetyltransferase, ChAT), transport (e.g., the vesicular ACh transporter, vAChT and the choline transporter, Cht1) and hydrolysis (e.g., acetylcholinesterase). Since NGF levels are directly related to the health and function of BFCNs [30], the high affinity neurotrophin receptor, TrkA, and the low affinity receptor, p75NTR, are also useful identifiers.

Due to their extraordinary complexity, the characterization of the complete morphology of cholinergic neurons has been difficult. In vitro studies from primary cultures showed that BFCNs have large cell bodies with two to four primary neurites [33]. However, a recent study in mice has demonstrated that individual cholinergic neurons from the basal forebrain region have axons that develop up to 50 cm in length, with approximately 1000 branches [34]. The electrophysiological profile of BFCNs has been characterized in rats, where BFCNs show regular spontaneous discharge patterns with mean spontaneous activity of 20 impulses/s [35]. Further key characteristics of the BFCNs are a slow spiking activity (4–10 Hz) and slow after potentials (400–700 ms) when compared with non-cholinergic neurons (3–60 ms) [36], which have also been shown in iPSC-derived BFCNs [37]. The addition of NGF can increase slow depolarization and enhance synaptic activity by upregulating ChAT activity [38].

#### Current differentiation strategies for cholinergic neurons

**Fig. 1:**
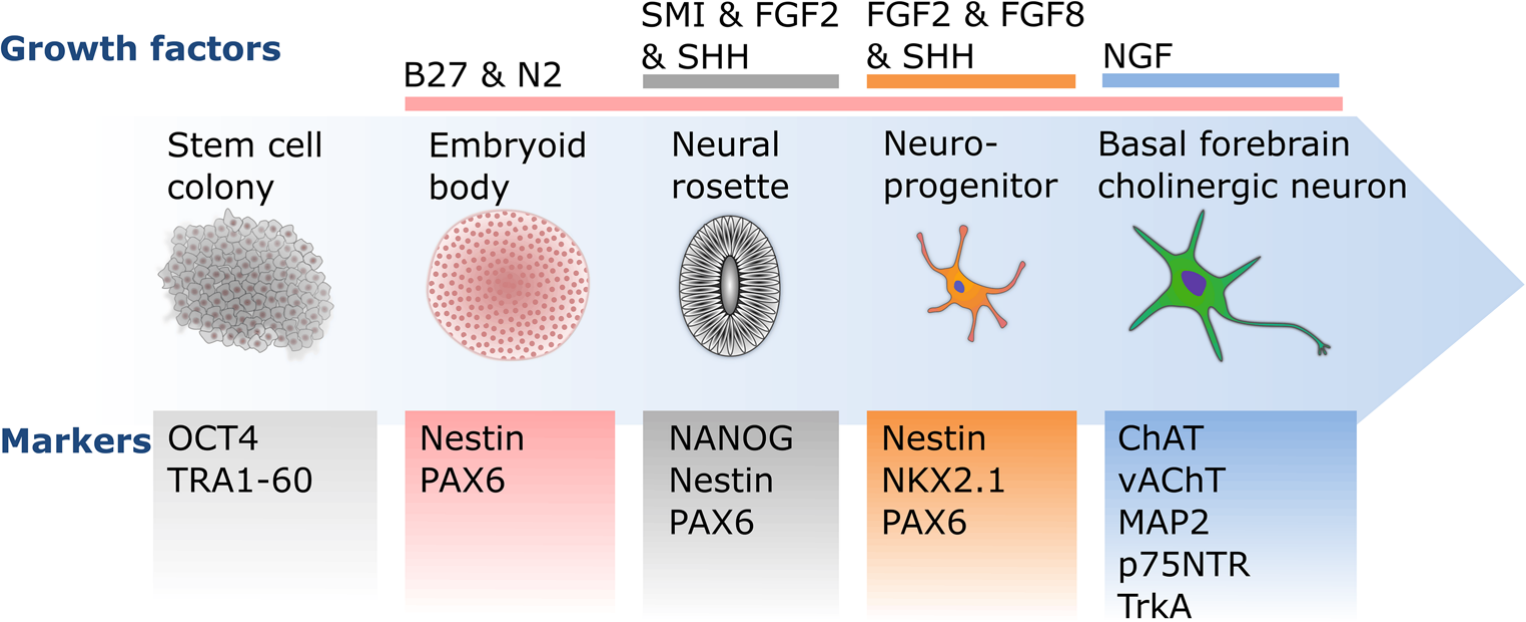
Schematic diagram of basal forebrain cholinergic neuron differentiation. The differentiation of basal forebrain cholinergic neurons from pluripotent stem cell colonies is driven by transitions between two- and three-dimensional culturing stages, as well as timed exposure to essential growth factors, such as NGF. The presence or absence of developmental and maturation markers are essential guides to monitor the differentiation progress at each culturing stage towards mature, functional forebrain cholinergic neurons

Cholinergic neurons derived from human embryonic stem cells (ESCs) and iPSCs can become an important tool for modeling neurodegenerative diseases, such as Alzheimer’s disease. However, due to their complexity, few studies have successfully differentiated BFCNs. Most of these studies start the differentiation process with the generation of embryoid bodies, followed by the formation of neural rosettes and neurospheres. These neurospheres contain neural precursor cells (NPCs) from which the different protocols will generate the BFCNs (Fig. 1).

Nilbratt et al. assessed growth factors that could induce forebrain identity [39]. BDNF, NGF, ciliary neurotrophic factor (CNTF) and neurotrophin 3 (NT-3) were tested as candidates, but only BDNF and NGF reliably induced the expression of both NKX2.1 and LHX8 [39]. Following this, Bissonnette et al. [32] published a comparison of two different protocols to generate functional BFCNs from hESC. In both cases cells were pre-treated with RA, SHH and FGF8 [32]. One option relied on diffusible ligands for differentiation (Table 1), while the other additionally transfected an expression plasmid encoding the transcription factors *LHX8* and *GBX1* and a fluorescent tag. Transfected cells could be purified by fluorescence activated cell sorting, a step that increased the ratio of BFCNs in the final culture to 94 %. This method has also allowed the successful differentiation of BFCNs derived from iPSCs, as described by the same group, showing that the iPSC-derived BFCNs can be used as a model for Alzheimer’s disease, producing disease-related pathological features [40].

**Table 1:** Comparison of basal forebrain cholinergic neuron differentiation protocols

Differentiation protocol	Nilbratt et al. [39]	Bissonnette et al. ([32], BMP9 treatment)	Bissonnette et al. ([32], nucleofection), Duan et al. [40]	Liu et al. [41]	Crompton et al. [37]
Duration (days)	ND	34	34	45	90
Efficiency (ChAT^+^ cells)	69–78 %	85 %	65 %; 94 % after FACS purification	38 %	>90 %
Growth factors	BDNF, CNTF, EGF, FGF2, NGF, NT-3	BMP9, EGF, FGF2, FGF8, NGF, RA, SHH	EGF, FGF2, FGF8, NGF, RA, SHH	BDNF, BMP9, cAMP, IGF-1, NGF, SHH	EGF, FGF2, SMI
Developmental markers (protein, mRNA)	BF-1, DLX1, DLX2, GBX2, GSH2, ISL1, LHX8, MASH1, NKX2.1	FORSE1	FORSE1, FOXG1, MASH1, NKX2.1	FOXG1, ISL1, MASH1, NKX2.1, OLIG2	FOXG1, ISL1, LHX8, NKX2.1
Maturity markers (protein, mRNA)	ChAT, nAChRs, NMDAR, mAChRs, MAP2, p75NTR, TrkA, β-III-tubulin	AChE, Calbindin, ChAT, MAP2, p75NTR, TrkA, vAChT	ChAT, MAP2, p75NTR, vAChT	ChAT, p75NTR, SYN-1, β-III-tubulin, vAChT	ChAT, MAP2, p75NTR, SYN-1, β-III-tubulin, vAChT
Physiological function	Ca^2+^ response to ACh	ACh production and release	ACh production and release Functional voltage-gated channels	Spontaneous action potentials	ACh production and release Functional voltage-gated channels and cholinergic receptors Spontaneous action potentials

Using a different strategy, Crompton et al. published a protocol for non-adherent differentiation of iPSCs into BFCNs [37]. In this procedure, neurospheres were treated with Nodal/transforming growth factor beta (TGF-β) inhibitor (small molecule inhibitor, SMI) to induce the endogenous expression of SHH, instead of its direct addition, resulting in a 90 % efficiency of β-III-tubulin/ChAT-expressing cells after 90 days [37].

Overall, only two of the mentioned protocols successfully reached >90 % ChAT-expressing cells. The main differences between the protocols are in their way of culturing (i.e., adherent by Bissonnette et al. [32] versus non-adherent by Crompton et al. [37]). This highlights the need for independent replication of both protocols to provide evidence for the use of either strategy. One potential advantage of the protocol developed by Bissonnette et al. involves using plasmid transfection via electroporation to trigger BFCN differentiation [32]. While this step allows fluorescently tagged cell sorting for purified cultures, transfection efficiency likely differs between each stem cell line and thus requires thorough optimization and counting of viable cells after sorting to produce replicable cultures.

In summary, the majority of published protocols for cholinergic differentiation are based on the initial addition of SHH or its endogenous induction to induce ventral forebrain fate and the expression of developmental markers of the MGE. While treatment with NGF has been shown to be highly important for the generation of mature ChAT-expressing BFCNs (Fig. 1; Table 1), the incomplete functional characterization of mature BFCNs limits us from recommending a particular protocol. This shortcoming can be addressed by transplanting BFCN precursors into rodents to compare the in vitro maturation with in vivo maturation of cells from the same origin. While three of the listed protocols show that engrafted BFCN precursors develop into integrated BFCNs [32, 37, 41], none of the studies compared the in vitro differentiated cells with their in vivo counterparts. We can, thus, not yet recommend a reliable BFCN differentiation protocol.

### Midbrain dopaminergic neurons

Midbrain dopaminergic (mDA) neurons are predominantly expressed in the substantia nigra pars compacta (SNc) and the ventral tegmental area (VTA) in rodents and primates [42–44]. SNc mDA neurons are required for initiation and control of motor functions, while VTA mDA neurons are important for reward behavior and cognition. Both nuclei are implicated in severe disorders, with degeneration of SNc mDA neurons being a hallmark of Parkinson’s disease, and impaired signaling of VTA mDA neurons being implicated in psychiatric disorders, such as schizophrenia and bipolar disorder. There is thus strong interest in differentiating human mDA neurons in vitro to study mechanisms contributing to the onset and progression of these disorders.

#### Mammalian development of mDA neurons

Midbrain DA neurons arise from NPCs of the ventral mesencephalon in mammals. The expression of aldehyde dehydrogenase 1 by progenitor cells at embryonic day 9.5 (E9.5) in mice triggers the development of post-mitotic cells, which produce the dopamine synthesizing enzyme tyrosine hydroxylase (TH) [45]. These mDA neuron precursors express nuclear receptor related 1 protein (NURR1) at E10.5 and differentiate into dopamine producing, TH-expressing neurons at E11.5 in the mediobasal floor plate [45, 46]. Neurogenesis of mDA neurons peaks at E12.5 in mice and E80 in non-human primates [42, 47–49], with mDA neuronal development occurring earlier in the SNc than VTA [47]. While the timing of prenatal mDA neurogenesis is similar in mice and rats, neurite development and migration is completed in postnatal week one in mice and three in rats [46, 50]. This suggests a likely further extension to the developmental period in primates that has yet to be mapped in humans.

Neurogenesis of mDA neurons is driven by intrinsic and extrinsic signaling factors with precise temporal release patterns. The specific events have recently been reviewed in detail [51, 52]. Briefly, mDA progenitor cells receive target-derived neurotrophic factors from the mesencephalon floor plate of the dorsoventral neural tube axis (SHH and FGF8) [53–55] and later from the striatum (glial cell-derived neurotrophic factor, GDNF and neurturin, NRTN) [56–58]. Additional factors, such as BDNF, TGF3β, RA and ligands for members of the frizzled family of seven transmembrane receptors (Wnt1 and Wnt5a), promote mDA neuronal development and midbrain organization [59–63]. The variations in developmental timing of mDA neurons between species suggest an importance for the scheduling of the different factors between stem cells of different organisms.

#### Characteristics of mature mDA neurons

Midbrain DA neurons develop into a distinct cell type throughout their differentiation and maturation process which can be identified through functional, morphological and protein expression characteristics. The functional profile of mDA neurons requires the production of several key proteins, which have therefore been used as markers to identify mDA neurons within brain regions and in mixed primary cultures. The dopaminergic profile ultimately relies on the synthesis of dopamine from l-3,4-dihydroxyphenylalanine (l-DOPA) by TH, the re-uptake of dopamine from the synaptic cleft by dopamine transporters (DAT1), as well as the auto feedback loop, regulating dopamine production through activation of presynaptic dopamine receptor 2 (DR2).

Promoter-driven expression or antibody labeling of TH has consequently been used to identify DA neurons in post mortem tissue [64], primary cell cultures [65] and human stem cell derived differentiated neurons [66]. However, TH is required for the first synthesis step of catecholamines and therefore also present in noradrenergic cells [67]. In addition, TH expression is tightly regulated by neuronal activity via modulation of histone acetylation levels and is neither specific to DA neurons nor expressed at consistent levels in these cells [68]. Selection based on DAT expression has shown high specificity for mature mDA neurons from the VTA, with lower expression levels in the SNc [69]. Consequently, purifying embryonic mouse brain cultures for DAT resulted in a higher proportion of mature mDA neurons than selecting for TH [70, 71]. To address limitations of TH and DAT expression based selection, a recent study explored cell surface proteins with high specificity for mDA neurons. Ganat et al. identified the nicotinic ACh receptor subunit 3 and 6 to be highly colocalized with mDA neurons in the mouse [72], suggesting that these receptor subunits could be additional selection options for stem cell-derived mDA neurons [73], following confirmation in human samples.

The morphological profile of mDA neurons in vivo is dominated by their long projections, connecting the VTA with the neocortex via the mesocortical pathway and the SNc with the striatum via the nigrostriatal pathway. Limitations of the in vitro environment restrict this structural characteristic, moving the focus onto cell body size and shape. Midbrain DA neurons in the VTA are reportedly smaller and rounder than their elongated SNc relatives (13 vs. 19 µm Ø) [74]. Additionally, VTA mDA neurons are predominantly multipolar with radial projections while SNc mDA neurons have largely lateral and ventral projecting dendrites [75]. Although the reported characteristics are based on rodent in vitro and in vivo studies, they are expected to be observed in stem cell derived mDA neurons [76].

The electrophysiological profile of mDA neurons has been characterized in vivo and in vitro, identifying specific action potential patterns. Midbrain DA neurons can express either high bursts (>15 Hz) or slow background (1–5 Hz) action potential discharges [64], with each discharge starting with a prominent hyperpolarizing pulse [77] and depend on the G-protein-regulated inward-rectifier potassium channel 2 (GIRK2). The burst pattern has been shown to depend on the morphology of the mDA neurons [78] and requires specific stimulation in vitro [79]. This suggests that the functional assessment of stem cell-derived mDA neurons by electrophysiological characterization is met with several challenges, as the full phenotype depends on the input of the surrounding network and extracellular concentration of dopamine [77]. However, as similar action potential patterns have been identified in iPSC-derived mDA neurons [73], the electrophysiological profile forms the third critical characteristic of in vitro differentiated mDA neurons after protein expression and dopamine handling.

#### Current differentiation strategies for mDA neurons

**Fig. 2:**
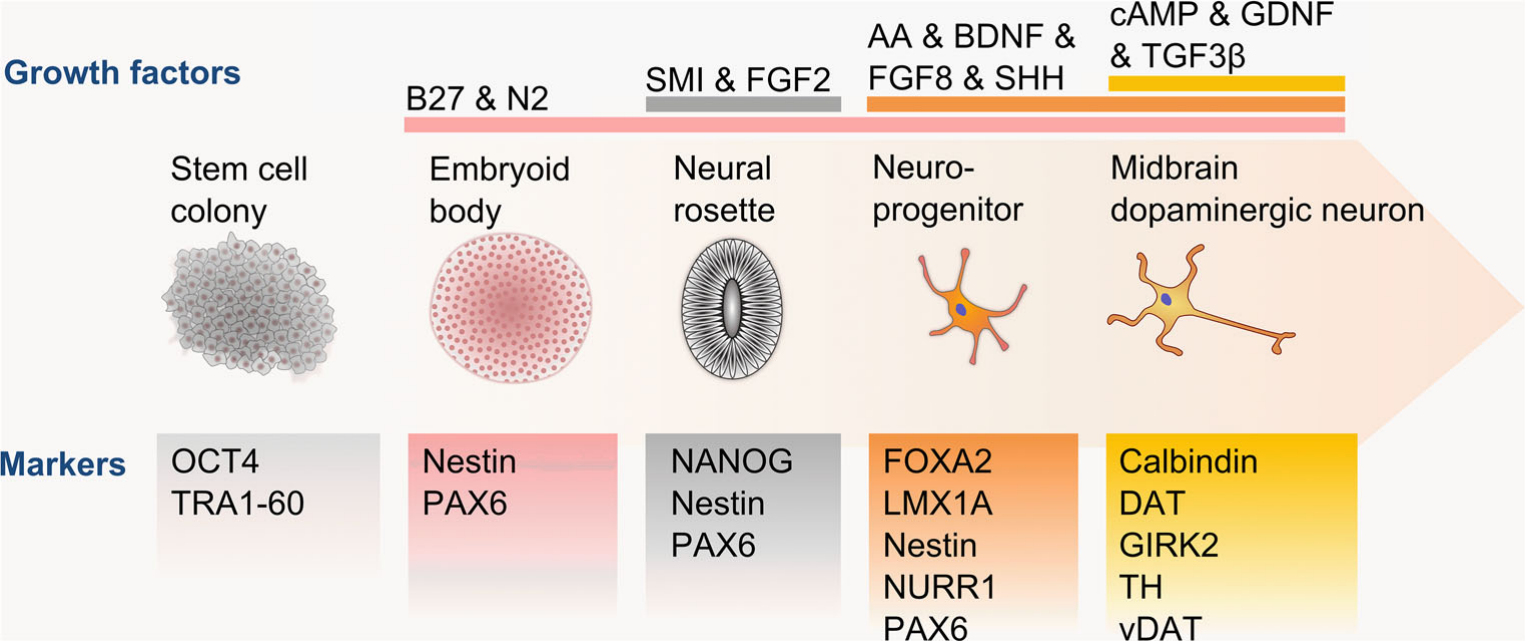
Schematic diagram of midbrain dopaminergic neuron differentiation. The differentiation of midbrain dopaminergic neurons from pluripotent stem cell colonies is driven by the transition from spherical to adherent cultures and a staggered supplementation of growth factors, particularly SHH, FGF8 and later TGF3β. The presence or absence of developmental and maturation markers are essential guides to monitor the differentiation progress at each culturing stage towards mature, functional midbrain dopaminergic neurons

The strong disease relevance of mDA neurons and their distinct developmental pathway has driven the differentiation of mDA neurons in vitro. Following the availability of mouse NPCs, mouse and human ESCs and more recently iPSCs, different strategies have been developed to create in vitro cultures rich in mature mDA neurons (Table 2). The majority of differentiation protocols closely follow the in vivo developmental stages, progressing from stem cells to neural rosettes (sometimes via embryoid body formation) to create neural progenitor cells and begin the mDA neuron patterning (Fig. 2). Cells at each stage are supplemented with a cocktail of growth factors reportedly found during the relevant developmental stages, such as SHH, FGF8 and BDNF (Table 2). Despite extensive testing of variations in media compositions, including undefined commercial options, and differentiation timings, the reported efficiency ratios have remained low with 8–40 % of generated cells expressing TH (Table 2). Higher yields are promised by the use of SMI, specifically targeting Wnt and GSK3β signaling [80] and factors involved in floor plate development [81]. However, since GSK3β signaling is tightly regulated during neurodevelopment [82], the risk of unintended consequences on mature mDA neurons remains to be assessed. To ensure the formation of physiologically relevant cells, the majority of protocols already go beyond TH expression analysis to identify mature mDA neurons. Critically, the thoroughness of each protocol in identifying the derived cultures still varies widely (Table 2), with two protocols presenting TH expressing cells without stating their proportion within the differentiated cultures [80, 83]. By not reporting electrophysiological characteristics, for example, it remains unclear whether the created dopamine-producing cells are close representations of SNc, VTA or other dopamine neurons. The protocol by Kriks et al. [81] uses fully adherent differentiation to form a promising foundation for further development and optimization of mDA neuron differentiation, with the strong need for identifying the full spectrum of the generated cultures.

**Table 2:** Comparison of a representative selection of midbrain dopaminergic neuron differentiation protocols

Differentiation protocol	Brennand et al. [6]	Chambers et al. [80]	Fathi et al. [85]	Hartfield et al. [73]	Kriks et al. [81]	Perrier et al. [86]	Petit et al. [83]	Reinhardt et al. [84]	Tan et al. [87]
Duration (days)	110	19	30	66	50	62	90	28	28
Efficiency (TH^+^ cells)	8 %	ND (80 % PAX6^+^)	35 %	20.31 %	78 %	40 %	ND (75 % β-III-tubulin^+^)	35 %	33 %
Growth factors	AA, BDNF, dcAMP, FGF2, GDNF	AA, BDNF, cAMP, FGF2, FGF8, GDNF, Noggin, TGF3β	AA, BDFN, dcAMP, FGF2, GDNF, LMX1A, SHH, SMI	AA, BDNF, dcAMP, FGF8, GDNF, Noggin, SHH, SMI	AA, BDNF, CHIR, DATP, dbcAMP, FGF8, GDNF, Purmorphamine, SHH, SMI, TGF3β	AA, BDFN, dcAMP, FGF2, FGF8, GDNF, SHH, SMI	AA, BDNF, cAMP, FGF8, GDNF, Noggin, SHH, SMI, TGF3β	AA, BDNF, CHIR, dcAMP, FGF8, GDNF, PMA, SMI, TGF3β	FGF2, FGF8, SHH
Developmental markers (protein, mRNA)	Nestin	CDX2, FGF5, Nestin,OTX2, PLZF, PAX6, SOX1, SOX17	EN1, FOXA2, LMX1A, LMX1B, Nestin, PITX3, TAT, WNT1	FOXA2, Nestin, NURR1, PAX6	FOXA1, FOXA2, HESS, LHX2, LMX1A, NURR1, OTX2, PAX6	CRIPTO, NCAM, Nestin, PAX6, SOX1	DNMT3B, FOXA2, Nestin, PAX6	Brachyury, FOXA2, Nestin, PAX6, PAX7, SOX1, SOX9, TFAP2A	NURR1
Maturity markers (protein, mRNA)	GEPH, GluR1, MAP2, PSD95, SYN-1, TH, β-III-tubulin, VGAT, VGLUT1	TH, β-III-tubulin	MAP2, PITX3, TH	AADC, Calbindin, DAT1, GBA, GIRK2, IP3R, LRRK2, PITX3, α-synuclein, tau, TH	DAT, GIRK2, TH, β-III-tubulin	AADC, EN1, GFAP, MAP2, O4, PAX2, PAX5, SV2, SYN-1, TH, VMAT2	TH, β-III-tubulin	MAP2, PAX3, PAX7, TH, β-III-tubulin	ALDH1A1, GFAP, MAP2, PITX3, SCN1A, TH, β-III-tubulin, VMAT2
Physiological function	DA production, spontaneous Ca^2+^ oscillations, spontaneous EPSPs and IPSPs	ND	ND	DA production and uptake, spontaneous Ca^2+^ oscillations, mDA-specific mitochondrial responses, slow action potential trains	DA production, slow action potential trains and spontaneous spikes	DA production, repetitive action potential trains	DA production	Spontaneous action potentials	ND

An important shortcoming across all mDA neuron differentiation protocols still remains: the direct comparison of in vitro differentiated neurons with engrafted and in vivo differentiated neurons. Two of the discussed protocols engrafted differentiated NPCs [84] or mDA neurons [81] into rodents, showing that these cells integrate and mature in their host environment. Neither of the protocols, however, compared the matured in vitro cultured cells with their in vivo relatives. The extent to which stem cell derived mDA neurons differ to their in vivo counterparts thus remains unclear. This information is vital to assess whether stem cell derived mDA neurons are appropriate for the range of questions they have been promised to answer.

### Cortical astrocytes

Once defined as the “nerve glue” (neuroglia) for neurons, astrocytes have emerged as one of the key players in maintaining cellular homeostasis in the brain and spinal cord. Astrocytes regulate the ion concentration and remove excess neurotransmitter and cellular debris from the extracellular fluid surrounding neurons. In neurodegenerative diseases, however, this vast support network for neurons is often altered or dysregulated [88]. The dysregulation of astrocytes can lead to excessive neuroinflammation, a common pathology in neurodegenerative diseases, and contributes to neuronal deterioration [89]. It is, therefore, important to study the contribution of astrocytes to neuronal degeneration and disease.

#### Mammalian development of astrocytes

Astrocyte generation and maturation in vivo relies on precise temporal and positional stimuli provided by the cellular environment and neighboring cells [90]. In mice, the differentiation of astrocytes and other neural cells begins at E8.5 with neurulation of the neuroectoderm [91]. Neuroepithelial cells from the neuroectoderm firstly differentiate into radial glia, the precursors of astrocytes and NPCs [92]. Although radial glia and astrocytes are overlapping in their marker expression of proteins, such as vimentin and glial fibrillary acidic protein (GFAP) [93], the morphology, function and time of development differ significantly. In the mammalian brain, the main morphological characteristic of radial glia is their long processes, which extend from the ventricular zone all the way to the marginal zone of the pial surface [94]. Through asymmetric horizontal division, radial glia give rise to NPCs and initiate neurogenesis. The newly formed NPCs then migrate along the long processes of radial glia to form the different layers of the cortex [95]. In the late prenatal stage and early postnatal stage (E18-P7) in mice, after neurogenesis and neuronal migration are completed, radial glia differentiate into astrocytes, a process called astrogliogenesis.

The differentiation of radial glia and NPCs into astrocytes is dependent on the activation of several signaling pathways. Two of these pathways are the JAK/STAT and the BMP-SMAD pathway [96]. These pathways are extrinsically activated by growth factors, including CNTF, cardiotrophin 1 (CT-1) and leukemia inhibitory factor (LIF), which are released by early neurons and late NPCs in vivo. Activation of these mechanisms leads to downstream events including chromatin modification and induction of astrocyte specific gene expression [97]. Transcription factors, such as signal transducer and activator of transcription 3 (STAT3), mothers against decapentaplegic homolog 1 (SMAD1), SMAD4 and nuclear factor 1A (NF1A) are activated and initiate the expression of GFAP, S100 calcium binding protein B (S-100β) and glutamate aspartate transporter (GLAST), all of which are currently used as astrocyte specific markers [98–100]. Within the first week after birth, astrocytes develop branched processes and attain their star like structure in the postnatal brain and spinal cord in mice [101]. Moreover, expression of the mature astrocyte marker glutamate transporter 1 (GLT1), a membrane protein important for the protection of neurons from glutamate-mediated excitotoxicity, was observed 2–3 weeks after birth [102]. Mouse studies have further shown that developmental GLT1 expression is upregulated in vitro when astrocytes are co-cultured with neurons [103]. This indicates that neuronal signaling is important for astrocyte maturation.

To successfully differentiate stem cells into functionally and physiologically relevant astrocytes, the molecular pathways involved in the cell fate determination of astrocytes in vivo need to be replicated in vitro. However, as most of the developmental studies are based on mouse models, the translation of this information to human stem cell differentiation requires careful consideration.

#### Characteristics of mature astrocytes

In the pursuit of generating functional astrocytes, it is essential to assess the differentiated cells for the presence of astrocyte-specific characteristics that are generally observed in vivo. Morphologically, early astrocytes have large cell bodies with few processes. However, as astrocytes mature, more processes develop, elongate and branch out, giving the typical ‘star’ shape. In mice, this branching and elongation of processes takes place during the late postnatal stages (P14–P27) [104], suggesting that the astrocyte networks are formed after the maturation of neighboring neurons.

On the molecular level, the expression of a selection of proteins is commonly used to characterize cells, in combination with cellular morphology. The most widely used marker in the characterisation of astrocytes is GFAP. In vivo mouse studies have shown that GFAP expression during astrocyte maturation peaks between E16-P1, slowly decreasing after this time point [105]. Mature astrocytes only express low levels of GFAP; however, GFAP expression in mature astrocytes may be upregulated following inflammatory activation [106]. Hence rather than being a marker for mature and functional astrocytes, GFAP expression is more indicative of either early immature astrocytes or reactive astrocytes. Another early developmental astrocyte marker is NF1A, an astrocyte specific transcription factor. This protein is highly expressed during mouse embryonic development (E10–E12.5), but its expression decreases as astrocytes mature [100]. Conversely to GFAP and NF1A, the expression of the astrocyte specific proteins aldolase C (ALDOC), GLAST and GLT1 increase as astrocytes mature [102, 107, 108]. These proteins therefore provide more appropriate markers of mature astrocytes than the commonly used GFAP. In humans, expression of the GLAST and GLT1 homologues, excitatory amino acid transporter 1 (EAAT1) and EAAT2, respectively, also increases with gestation time, based on studies of post mortem fetal brain tissue [109]. As such, increases in EAAT1 and EAAT2, coupled with a decrease in the early astrocyte markers, GFAP and NF1A, could be used as a robust indication of astrocyte maturation.

Other astrocyte specific protein markers commonly chosen are S100β, aldehyde dehydrogenase 1 family member L1 (ALDH1L1) and the surface marker protein, CD44. The expression of these proteins increases at approximately E13 in mice and the expression profile does not significantly change throughout development [110–113]. Thus, while being astrocyte specific markers, these proteins are unsuitable indicators of astrocyte maturity.

Although the expression profile of astrocyte-specific proteins provides important information regarding their maturity, it does not offer information about their functionality. Astrocytes provide a support network for neurons by closely monitoring and responding to the extracellular environment. One of the main functions of astrocytes is protecting neurons from excess neurotransmitter stimulation, by taking up glutamate via GLT1 and GLAST [114]. Additionally, astrocytes are a key player of the innate immune system of the CNS. As such, they express toll-like receptors (TLRs), which are able to recognize foreign particles [115]. Activation of TLRs leads to the release of chemical signaling molecules, such as cytokines and chemokines [e.g., interleukin-6 (IL-6), interferon-β and fractalkine (CX3CL1)] [116]. These molecules recruit and activate other immune cells to the site of injury. Furthermore, activated astrocytes also release neurotrophic factors, such as NGF, CNTF and BDNF to aid neuronal survival and regeneration. The controlled release of these immune and neurotrophic factors is thus a critical aspect of astrocyte function, which needs to be confirmed during in vitro differentiation.

Overall, a panel of marker proteins should be used to identify the presence of mature astrocytes. Verifying their functional profile, particularly immune response and neurotransmitter handling, will ensure the relevance of in vitro astrocytes for modeling disease-relevant processes.

#### Current differentiation strategies for astrocytes

The generation of mature astrocytes for the purpose of studying neurological diseases has led to the development of several astrocyte differentiation protocols from pluripotent stem cells. However, these protocols vary in their duration, growth factor conditions and efficiency (Table 3).

**Table 3:** Comparison of astrocyte differentiation protocols

Differentiation protocol	Krencik et al. [117]	Emdad et al. [118]	Serio et al. [119]	Shaltouki et al. [120]	Roybon et al. [122]	Mormone et al. [121]
Duration (days)	120	35	49	42	90	35
Efficiency (GFAP^+^ cells)	90 %	70 %	90 %	70 %	70 %	55 % (without sorting)
Growth factors	CNTF (or LIF), EGF, FGF2	CNTF, EGF, FGF2	CNTF, EGF, FGF2 LIF	CNTF, FGF2, NRG1β1	AA, BDNF, CNTF, FGF2, GDNF, IGF, RA; maturation induced by withdrawal of growth factors	CNTF, EGF, FGF2
Developmental markers (protein, mRNA)	CD44, GFAP, NF1A	GFAP	GFAP, NF1A, vimentin	CD44, GFAP, NF1A	AQP4, CD44, GFAP, NF1A, S100β, vimentin	GFAP
Maturity markers (protein, mRNA)	S100β	AQP4, EAAT1	EAAT1, S100β	ALDOC, EAAT1, S100β	ALDH1L1, EAAT1, EAAT2	ALDOC, EAAT2
Physiological function	Propagation of Ca^2+^ waves, glutamate uptake	Migratory properties	Glutamate uptake, promotion of synaptogenesis in neuron co-cultures	Glutamate uptake, promotion of synaptogenesis in neuron co-cultures	Propagation of Ca^2+^, glutamate uptake, inflammatory response (IL-6 release)	Migratory properties

**Fig. 3:**
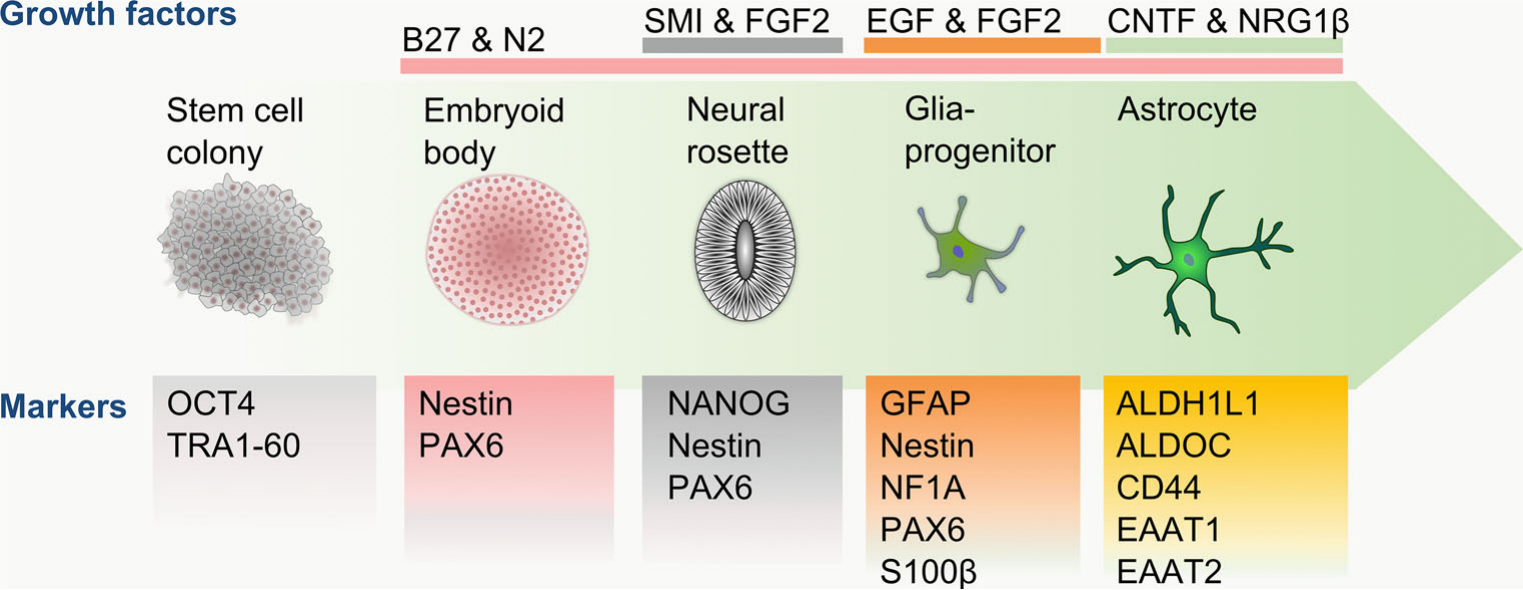
Schematic diagram of astrocyte differentiation. The differentiation of astrocytes from pluripotent stem cell colonies follows the early neuronal developmental progress through spherical and adherent culture stages. Glial progenitor formation is triggered by the supplementation of EGF and FGF2, with CNTF being required for transition to mature astrocytes and aided by the neurotrophic factor NRG1β. The presence or absence of developmental and maturation markers are essential guides to monitor the differentiation progress at each culturing stage towards mature, functional astrocytes

The generation of neural rosettes and NPCs via embryoid body formation is similar across all protocols, lasting approximately 21 days. However major variations between protocols arise during the differentiation and maturation of NPCs to functional astrocytes. Once NPCs are present, the most common treatment in the generation of astrocytes is supplementation with epidermal growth factor (EGF) and FGF2 (Fig. 3), leading to the generation of early astrocytes, which has been confirmed by the expression of CD44, NF1A and vimentin [117–121]. Once the presence of early astrocytes is confirmed, the maturation of these cells is generally triggered by the addition of CNTF (Fig. 3). Although the growth factor conditions are very similar for most of the protocols (Table 3), a major difference lies in the maturation duration between each method. While Krencik et al. [117] matured the differentiated astrocytes for 100 days, the maturation time described by Emdad et al. [118], Serio et al. [119], Shaltouki et al. [120] and Mormone et al. [121] vary between 14 and 21 days. The discrepancy in maturation time was reflected in the proportion of GFAP-expressing cells, which varied between 55 % [121] and 90 % [117, 119]. To validate the functionality of the generated cells, some of these studies also confirmed the ability of astrocytes to take up glutamate from the cell medium [117, 119, 120].

Unlike the previously described astrocyte differentiation protocols, Roybon et al. [122] used a different approach in differentiating astrocytes. The generated NPCs were not directly differentiated into astrocytes; instead the cells were first caudalized using RA and ascorbic acid (AA), prior to treatment with neurotrophic factors, including CNTF, to generate neurons. The differentiation of astrocytes was induced by the withdrawal of neurotrophic factors and the introduction of foetal bovine serum. The generated astrocytes were matured for 50–90 days. While previous protocols assessed the maturation of cells with the expression of GFAP, Roybon et al. [122] used this protein as a marker for early astrocytes and EAAT1, EAAT2 and ALDH1L1 to identify mature astrocytes. Moreover, the differentiated astrocytes took up glutamate and released IL-6 upon stimulation with tumor necrosis factor-α and IL-1β [122]. This study provides initial evidence that the differentiated cells are able to mount an immune response; however, the complexity of this activation requires further characterisation.

Further evaluations of astrocyte maturation and function were conducted by transplanting pluripotent stem cell derived astrocytes into mice brains [117, 120–122]. These in vivo experiments primarily demonstrated that transplanted cells retained their astrocyte identity as indicated by the expression of GFAP. Krencik et al. demonstrated that transplanted differentiated astrocytes were able to form direct contact with cerebral blood vessels after 6 months, suggesting that in vitro derived cells are able to mature in vivo towards functional astrocytes [117]. Conversely, however, Roybon et al. analyzed marker expression of engrafted astrocytes in vivo and found high expression of immature markers, such as NF1A [122]. Thus, even in vivo transplantation of in vitro generated astrocytes may not be able to promote full maturation of these cells. Further characterisation of in vitro differentiated astrocytes is therefore essential.

In summary, current astrocyte differentiation protocols use growth factors aligned with reported in vivo development. The heavy reliance on GFAP as an indicator for differentiation success and the limited reporting of functional characteristics prevent a reliable assessment of the maturity of the created cells. Furthermore, the differentiation periods vary vastly between the protocols, highlighting the uncertainty about the required phenotype of the generated astrocytes for relevant experimentation. To date, the protocol described by Krencik et al. [117] (and Serio et al. [119]) has reported the highest proportion of GFAP^+^ cells (90 %) with the confirmation of physiological functions, such as the propagation of Ca^2+^ waves and glutamate handling, following a labor-intensive maturation duration of 4–6 months. Roybon et al. reduced the maturation time of pluripotent stem cell derived astrocytes to 90 days, while retaining a similar efficiency [122]. This protocol [122] relies on several markers to track the maturity of the generated astrocytes and reports the most extensive physiological confirmation by showing the propagation of Ca^2+^, glutamate handling and inflammatory response upon activation. For the purpose of accurate disease modeling, the protocol by Roybon et al. shows the essential maturity confirmation necessary, consisting of a thorough expression analysis of maturity markers (EAAT1, EAAT2 and ALDOC), in conjunction with functional characterisation (neurotransmitter processing and inflammation response) relevant to the studied disease.

## Assessing differentiation techniques

### Checklist for high quality and reproducible differentiated cultures in vitro

Induced pluripotent stem cells offer much promise in developing in vitro models to understand neurodegenerative disease mechanisms and for testing potential therapeutics. However, it is essential to generate a meaningful cell population that is both physiologically and clinically relevant. Evidently, an understanding of the desired cell type and its key characteristics is necessary to deliver high quality and reproducible cultures.

The following are common experimental considerations to promote cell culture purity and consistency when performing or assessing differentiation:

1. *Adjust culture environment for each differentiation stage*. The extracellular matrix (ECM) directly and indirectly affects the maintenance and differentiation of stem cell and neural cultures [123]. Human ESCs and iPSCs have been historically cultured on living feeder cells for mechanical and chemical stimulation, but recent research has shown that complex ECM protein combinations are superior in promoting culture stability and proliferation [124, 125]. The ECM dynamically evolves during neurodevelopment to form separate compartments in the CNS [126], comprised of different ECM protein combinations [127, 128]. Recapitulating in vivo neurodevelopment in culture thus requires adjustment of the ECM environment for the desired cell type and developmental stage. Differentiation of neocortical cells has shown to benefit from combining ECM proteins such as collagen I and fibronectin [129], but new differentiation protocols might require performing an ECM microarray [130]. Advanced three-dimensional culture surface modification can additionally be used to further the maturation of the desired cell type [87].
2. *Optimize cell counts and the maturation duration of the protocol*. Cell plating density affects differentiation, potentially due to alterations in effective local growth factor concentrations. Careful assessment of the appropriate cell density for plating needs to be optimized since different cell lines and different cell types proliferate at different rates. Longer protocols may be required for mature phenotypes but can lead to greater differences in the development of the required cell type compared to contaminating (unwanted) cell types. For example, developing neurons exit the cell cycle, whereas contaminating cells may continue to divide unless removed or their growth is inhibited.
3. *Identify checkpoints along the differentiation pathway and use a panel of cell markers*. Expression of appropriate markers at checkpoints should be confirmed; also consider the use of positive or negative selection of cells, based on cell surface marker expression. At the final stage of differentiation the expression of a robust panel of cell-specific markers should be assessed for each line.
4. *Develop reporters to assess differentiated cell types*. Reporters using fluorescent protein expression driven by an appropriate promoter can be used to identify cell types of interest. For example, motor neurons differentiated from ESCs have been identified in mixed cultures via lentiviral delivery of a construct bearing a GFP driven by the homeobox-9 promoter [131] and stable GFAP-driven TagRFP has been used to select iPSC-derived astrocytes [132]. Similar methods can be employed for other cell types using the promoter sequence of an appropriate cell-specific marker. Automated imaging options, such as the Incucyte live cell imaging system, can be used to provide images that are free from operator bias. Furthermore, fluorescent protein expression can also be used to sort the cells following differentiation, increasing the purity of the cell type of interest.
5. *Monitor the quality of cultures by assessing cellular functionality*. The expression of a panel of marker proteins provides some evidence that the appropriate cell type has been generated. However, there are very few proteins that are expressed in one cell type alone; many commonly used (and supposedly cell-specific) markers are often expressed in multiple cell types. Further tests are therefore required to confirm that functional cells have been generated. Functionality can be assessed using in vitro live cell assays and should focus on non-biased assessments, such as receptor ligand quantification in culture medium, prior to experimental use.

The steps involved in developing and optimizing a differentiation protocol are outlined in Box 1.
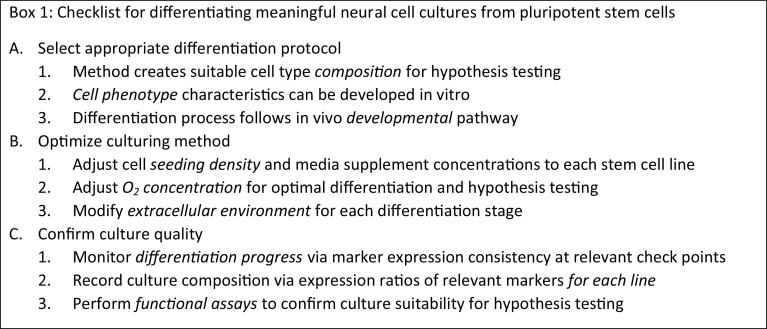


### Increasing the quality of cultures—suggestions for improvement

Cell culture experiments need to mimic the physiological environment as closely as possible, in order to yield biologically relevant conclusions. The following developments will allow the experimental set-up to more closely represent the in vivo cellular background:

1. *Use of a hypoxic chamber to prevent chronic oxidative stress*. The majority of cell culture experiments are performed under atmospheric oxygen conditions, i.e. 21 % O_2_. However cells in the brain and much of the body experience far lower levels, thought to range between 1 and 11 % O_2_ [133]. Recent data suggests that the process of reprogramming somatic cells to iPSCs is more efficient when performed under hypoxic conditions [134, 135]. Reprogramming requires a shift in cellular metabolism from oxidative to glycolytic conditions [10] and the hypoxia-inducible factors play a role in the coordination of these metabolic changes [136]. Comparisons of differentiation protocols under atmospheric vs. physiological O_2_ conditions are still limited. However, in the case of differentiation of cardiomyocytes, hypoxic culture increased differentiation yields by up to 1000-fold [137]. Measurements throughout the brain suggest that local oxygen tensions are heterogeneous in nature, exhibiting spatial and temporal differences depending on the microenvironment [138]. For example, neurons of the cerebral cortex experience a low oxygen field compared to venous O_2_ [138]. Neurons in such an oxygen environment are more sensitive to changes in cerebral blood flow, in the provision of oxygen and nutrients and are potentially more sensitive to oxidative stress. Investigations using in vivo imaging, for example by two-photon microscopy, allow the mapping of the partial pressure of oxygen at the μm/s resolution [139]. A detailed understanding of the local oxygen environment will allow us to provide in vivo conditions during in vitro experiments, an important consideration for further development. Future studies need to consider the implications of performing differentiation, and experiments, at atmospheric O_2_.
2. *Problems with feeding cycles could be partially overcome with microfluidics or bioreactors*. A consideration in cell differentiation is the consistent and appropriate provision of nutrients and growth factors. Traditional cell feeding cycles of every 24 or 48 h can lead to dramatic variations in nutrient delivery and gradients of trophic factors. During the course of a culture, the concentrations of glucose, growth factors and micronutrients can vary widely in the growth medium, along with the pH. The proliferation and differentiation of stem cells is drastically affected by growth medium supplements [140], with the outcome that under different nutrient conditions the same cell line could yield different phenotypes. Microfluidics can be used to manipulate the delivery of medium on the micrometer scale, allowing for more controlled temporal and spatial supply of nutrients [141]. Alternatively larger cell preparations could be cultured in bioreactors that stir the growth medium; agitation has led to higher yields of differentiated cells in some protocols [137]. The use of bioreactors that are engineered to cultivate three-dimensional cultures, under hypoxic conditions, provides a further promising development [142].
3. Development of accurate co-culture systems and three-dimensional cultures. The architecture of the brain is clearly a complex multicellular environment. The presence of diverse cell types in cultures can be detrimental when studying cell-specific effects. However, a mixture of cell types may be required to understand a particular cellular process, such as neuronal:glial interactions or neuroinflammation. In addition, other cell types may be required for appropriate maturation. For example, astrocytes are required for the maturation of functional synapses [143] and in organizing the neuronal extracellular matrix [144]. As differentiation protocols improve so does our capacity for generating the appropriate mix of cells that function together. A key aspect of many neurodegenerative disorders is that multiple cell types are affected. Being able to accurately model the system holistically would be a huge leap forward in understanding the role of cellular interactions in disease pathogenesis. The generation of specific neuronal and glial subtypes remains under-developed. To accurately model the brain for neurodegenerative disease research, future studies will need to address the issue of subtype specification, potentially incorporating single cell analysis. In the case of excitable cells, cell function can be analyzed by electrophysiological assessment, coupled with single cell reverse transcription PCR to characterize the molecular signature of the cells generated. Faithful recapitulation of the molecular profile of specific cell subtypes is an important goal for future research.

## Conclusions

Creating in vitro models of central nervous system disorders with human stem cells provides the medical research community with a powerful new research tool. To ensure the use of this option to its full potential, differentiation strategies need to be carefully planned and executed depending on the cell type desired and the experimental read-out. Confirmation of a robust panel of cell-specific markers, coupled with functional assays, is further required to provide evidence that the appropriate developmental pathway has been effectively recapitulated. Future studies need to focus on cultivating cells in more physiologically relevant environments by manipulating oxygen tension, overcoming issues with growth factor and nutrient gradients and developing multicellular and three-dimensional culture systems. Employing these quality improvement and control measures will lead to more reliable and reproducible results with strong clinical relevance.
